# On the Identification of Associations between Five World Health Organization Water, Sanitation and Hygiene Phenotypes and Six Predictors in Low and Middle-Income Countries

**DOI:** 10.1371/journal.pone.0170451

**Published:** 2017-01-26

**Authors:** Hugh Ellis, Erica Schoenberger

**Affiliations:** Department of Geography and Environmental Engineering, Johns Hopkins University, Baltimore Maryland, United States of America; Cardiff University, UNITED KINGDOM

## Abstract

**Background:**

According to the most recent estimates, 842,000 deaths in low- to middle-income countries were attributable to inadequate water, sanitation and hygiene in 2012. Despite billions of dollars and decades of effort, we still lack a sound understanding of which kinds of WASH interventions are most effective in improving public health outcomes, and an important corollary–whether the right things are being measured. The World Health Organization (WHO) has made a concerted effort to compile comprehensive data on drinking water quality and sanitation in the developing world. A recent 2014 report provides information on three phenotypes (responses): Unsafe Water Deaths, Unsafe Sanitation Deaths, Unsafe Hygiene Deaths; two grouped phenotypes: Unsafe Water and Sanitation Deaths and Unsafe Water, Sanitation and Hygiene Deaths; and six explanatory variables (predictors): Improved Sanitation, Unimproved Water Source, Piped Water To Premises, Other Improved Water Source, Filtered and Bottled Water in the Household and Handwashing.

**Methods and Findings:**

Regression analyses were performed to identify statistically significant associations between these mortality responses and predictors. Good fitted-model performance required: (1) the use of population-normalized death fractions as opposed to number of deaths; (2) transformed response (logit or power); and (3) square-root predictor transformation. Given the complexity and heterogeneity of the relationships and countries being studied, these models exhibited remarkable performance and explained, for example, about 85% of the observed variance in population-normalized Unsafe Sanitation Death fraction, with a high F-statistic and highly statistically significant predictor p-values. Similar performance was found for all other responses, which was an unexpected result (the expected associations between responses and predictors–i.e., water-related with water-related, etc. did not occur). The set of statistically significant predictors remains the same across all responses. That is, Unsafe Water Source (UWS), Improved Sanitation (IS) and Filtered and Bottled Water in the Household (FBH) were the only statistically significant predictors whether the response was Unsafe Sanitation Death Fraction, Unsafe Hygiene Death Fraction or Unsafe Water Death Fraction. Moreover, the fraction of variance explained for all fitted models remained relatively high (adjusted R^2^ ranges from 0.7605 to 0.8533). We find that two of the statistically significant predictors–Improved Sanitation and Unimproved Water Sources–are particularly influential. We also find that some predictors (Piped Water to Premises, Other Improved Water Sources) have very little explanatory power for predicting mortality and one (Other Improved Water Sources) has a counterintuitive effect on response (Unsafe Sanitary Death Fraction *increases* with increases in OIWS) and one predictor (Hand Washing) to have essentially no explanatory usefulness.

**Conclusions:**

Our results suggest that a higher priority may need to be given to improved sanitation than has been the case. Nevertheless, while our focus in this paper is mortality, morbidity is a staggering consequence of inadequate water, sanitation and hygiene, and lower impact on mortality may not mean a similarly low impact on morbidity. More specifically, those predictors that we found uninfluential for predicting mortality-related responses may indeed be important when morbidity is the response.

## Introduction

According to the most recent estimates, 842,000 deaths in low- to middle-income countries were attributable to inadequate water, sanitation and hygiene in 2012 [1]. This figure represented 58% of total deaths attributed to diarrheal disease which, in turn, constituted an estimated 1.5% of the total Global Burden of Disease (GBD) [1, 2]. It is a notable reduction from the estimated 88% of total deaths attributed to diarrheal disease related to inadequate WASH in 2000. Diarrheal deaths as a whole fell from an estimated 2.2 million in 2000 to 1.5 million in 2012 [1–4].

UNICEF and WHO in a report on progress towards meeting Millenium Development Goals (MDGs) state that the MDG for drinking water–a 50% reduction in the number of people without sustainable access to safe drinking water–was achieved by 2010, five years ahead of schedule. According to their figures, 91% of the world’s population now has access to improved drinking water [5]. Note that the term ‘improved’ does not necessarily mean ‘safe’ according to WHO standards [4, 6, 7].The UNICEF/WHO report places particular emphasis on the category of piped water to premises, which it describes as the highest level of service in drinking water supply. The report goes on to acknowledge that the MDG for improvement in sanitation–halving the number of people without access to basic sanitation–has been missed by some 700 million people [5].

There has been heartening progress. Nevertheless, it is plain that much remains to be done [8, 9]. The way forward, however, is still unclear. There are three principal difficulties. First, the data we have available is of uncertain reliability, coverage and comparability across countries. This is true both for the data on mortality and on the presence or absence of different categories of WASH. Many of the available numbers may be considerably out of date. For example, 21 sub-Saharan African countries conducted no household surveys in the years 2006–2013. It is likely that the most vulnerable are underrepresented, including people living in urban slums or in conflict zones [7, 10, 11]. Second, the data is unusually heterogeneous, deriving from a wide range of sources including censuses, national registers and household surveys. This adds to the difficulty of interpreting statistical results. Third, despite decades and billions of dollars of effort, our understanding of which kinds of WASH interventions are most effective in improving public health outcomes is still weak. The lack of adequate randomized controlled trials of different kinds of intervention has been widely commented upon [12, 13].

The World Health Organization (WHO) has made a concerted effort to compile the most comprehensive and reliable collection of information to date [1]. This data, along with analyses of the GBD data and with meta-analyses of prior studies, has been closely examined in a noteworthy series of papers published in the *Journal of Tropical Medicine and International Health* in 2014 [2, 3, 7, 14–16].

Clasen *et al*. describe the evolution of the GBD, including changes in methods, definitions and scope. They also describe an alternative approach to understanding the impact of WASH interventions, using population intervention modeling [14]. Prüss-Ustün *et al*. [2] estimate the burden of diarrheal disease that can be attributed to exposure to inadequate water, sanitation and hygiene based on exposure data and a related exposure-risk relationship. This is where the figures cited above were obtained. They further decompose the global estimate of 842,000 deaths into 502,000 deaths related to inadequate drinking water, 280,000 deaths related to inadequate sanitation and 267,000 deaths related to inadequate hand hygiene. For children under five, they estimate 361,000 preventable deaths due to WASH-related diarrhea, or 5.5% of deaths in that age group.

Freeman *et al*. [15] provide a meta-analysis of hand washing studies. They estimate that adequate hand washing may reduce the risk of diarrheal disease by 40%. However, when they adjusted for unblinded intervention studies, this figure declined to 23%. They also suggest that only 19% of the world’s population practices adequate hand hygiene [15]. Bain *et al*. estimate exposure to fecal contamination in different kinds of drinking water source based on household surveys and censuses. They combined this with a meta-analysis of 345 water quality studies. They estimate that 1.8 billion people worldwide drink water from contaminated sources, with at least 1.1 billion exposed to at least moderate risk (> 10 *E*. *coli* or thermotolerant coliform per 100 ml). They found that an estimated 10% of “improved” water sources may be high risk with at least 100 *E*. *coli* or thermotolerant coliform per 100 ml. Significantly, this category includes piped water supplies. They found also that people living in rural areas and people living in Africa and Southeast Asia were at higher risk from contaminated drinking water sources. They suggest that the sizeable improvements in mortality related to WASH and diarrheal disease in the GBDs may overstate the actual gains [3, 4, 6, 17]. Wolf *et al*. offer a comprehensive meta-analysis of studies investigating the effect of drinking water and sanitation improvements. The studies range in date from 1970 to 2013 and include randomized controlled trials, quasi-randomized trials and different kinds of observational study. They show that improvements in water supply and sanitation reduce the risk of diarrheal disease. Greater impacts were associated with filtering, high quality piped water and sewerage connection [16].

In a later meta-analysis, Clasen *et al*. [12] examined the health impacts of different kinds of water supply interventions, including Point of Use (POU) technologies. The latter are increasingly seen as a viable interim measure in very poor and/or rural areas where the timely implementation of well-managed piped water systems is highly unlikely [18]. The authors found little evidence to support the idea that improvements at the source (e.g., protected wells, standpipes) had any significant impact on the burden of diarrhea. They were cautiously optimistic about POUs. This study draws particular attention to the fragility of much of the data that is available to assess WASH interventions.

These are valuable findings. They confirm that inadequate WASH infrastructure is related to negative public health outcomes and improvements in these outcomes vary according to different types of interventions. The authors, nevertheless, offer a number of caveats concerning our ability to derive policy-relevant inferences from the data and urge efforts at better data collection. Empirical evidence concerning the effects of a range of interventions is also heterogeneous and fragmentary. Decades of efforts in the field have not resolved recurrent, policy-relevant questions such as whether improvements in water supply are effective in the absence of improved sanitation or in the absence of behavioral changes such as hand washing [12, 19–26]. A better sense of the relative importance and interactions of predictor variables is sorely needed to guide investment strategies in WASH interventions.

This paper reports on an analysis of the latest WHO data that takes a somewhat different approach than has been used to date. Our goals in this endeavor are two-fold:

1. To distinguish among predictors with a finer resolution to determine their relative importance;
2. To generate hypotheses about the data itself concerning, for example, the treatment of outliers or the possible interpretations of certain predictors such as “piped water to premises” and “other improved water sources”.

## Methods

### Data

The data used in this work are described in the 2014 WHO report: Preventing Diarrhoea Through Better Water, Sanitation and Hygiene—exposures and impacts in low- and middle-income countries [1]. It provides data on six predictors–Piped Water to Premises (PWTP); Other Improved Drinking-Water Sources (OIWS); Unimproved Drinking-Water Sources (UWS); Filtered and Bottled Water in the Household (FBH); Improved Sanitation (IS); and Handwashing (HW). The responses in our analyses all involved mortality and included Unsafe Sanitation Deaths (USD) Unsafe Hygiene Deaths (UHD), Unsafe Water Deaths (UWD), Unsafe Water and Sanitation Deaths (UWSD) and Unsafe Water, Sanitation and Hygiene Deaths (UWSHD). These responses are highly correlated (see §B in S1 File). We begin by focusing on one of the responses in the data—Unsafe Sanitation Deaths–in order to illustrate the steps needed to create good-fitting models, but then broaden the analyses by reporting selected results for all responses. Complete results for all responses can be generated using the input data and R scripts described in §C in S1 File and provided in S1 Data.

### Statistical Analysis

The statistical analysis method used in this study was ordinary least squares regression. General diagnostic tests to help determine the legitimacy of using linear regression models include Q-Q plots and Shapiro-Wilk for normality of residuals, component plus residual plots to check for linearity between responses and predictors, Durbin-Watson to detect autocorrelation of residuals, Breusch-Pagan for constant error variance, Variance Inflation Factor for predictor correlation, and Cook’s Distance and leverage plots for outlier detection [27]. Following Tukey and Mosteller’s bulging rule [27], logit and power response transformations were attempted as was square root predictor transformation [28]. Model predictive performance was assessed using k-fold cross validation. The procedure involves randomly partitioning the data into k groups (we used 10, but the results are fairly insensitive to k), fitting an OLS regression model using k-1 of the groups, then validating on the remaining group and computing mean square prediction error. The process is repeated many times to stabilize randomization effects–we used 1,000 as implemented in the R package DAAG [29]. We emphasize the importance of diagnostic tests and data transformations. Without careful attention paid to linear model justification, situations like those described in Bartram et al. [7] can render OLS-based results misleading and inappropriate. The issue at hand here is not inadequacy of linear models, but rather performing the transformations needed to apply them and assess their validity (e.g., see [27] chapter 4).

Significance levels for all results were adjusted to control for family-wise error rate following Bonferroni and the Holm-Bonferroni (H-B) step-down procedure [30] and for false discovery rate FDR following Benjamini and Hochberg (B-H) [31, 32]. We choose to report three methods reflecting different objectives in adjusting for multiple comparisons: (1) FWER for controlling the probability of one or more type I errors by adjusting the rejection criteria of each of the individual hypotheses or comparisons, with Holm-Bonferroni representing a more powerful but less conservative variant of the traditional Bonferroni adjustment; and (2) FDR because it has improved control over the number of rejected hypotheses than Family Wise Error Rate methods (e.g., Bonferroni-Holm). FDR-controlling procedures have greater power but at the cost of increased rates of Type I errors [33].

## Results

### Basic Descriptors

Descriptive statistics of the data were generated including untransformed predictor and response correlations, pairs plots, kernel densities of raw responses, kernel densities of predictors and responses scaled by population (see §B in S1 File). The responses are very highly correlated. The highest negative correlations exist between PWTP and OIWS, PWTP and UWS, and, IS and UWS. The highest positive correlation is for IS and PWTP. All response and predictor densities are, unsurprisingly, non-normal.

### Initial Regression Models

The first regression models included the full complement of six WHO predictors (PWTP, OIWS, UWS, FBH, IS, HW) with unsafe sanitation deaths (USD) as response. This model yielded poor performance, as did models for all responses. In the case of USD, it explained less than 2 percent of response variance (adjusted R^2^ = 0.01842) with no statistically significant coefficient estimates as shown in Table 1.

**Table 1:** Regression Results–Run001—All Countries (145) No transformations—Response is USD.

	Estimate	Std. Error	t value	Pr(>|t|)	Bonferroni	H-B	B-H
(Intercept)	62637	188731	0.332	0.740	1	1	0.897
PWTP	-56372	189568	-0.297	0.767	1	1	0.897
OIWS	-50108	189193	-0.265	0.792	1	1	0.897
UWS	-62498	188706	-0.331	0.741	1	1	0.897
FBH	-2652	4373	-0.606	0.545	1	1	0.897
IS	-7117	5995	-1.187	0.237	1	1	0.897
HW	1720	13209	0.13	0.897	1	1	0.897

Residual standard error: 9952 on 138 degrees of freedom.

Multiple R-squared: 0.05932, Adjusted R-squared: 0.01842.

F-statistic: 1.45 on 6 and 138 DF, p-value: 0.1999.

### Scaled Response

**Fig 1:**
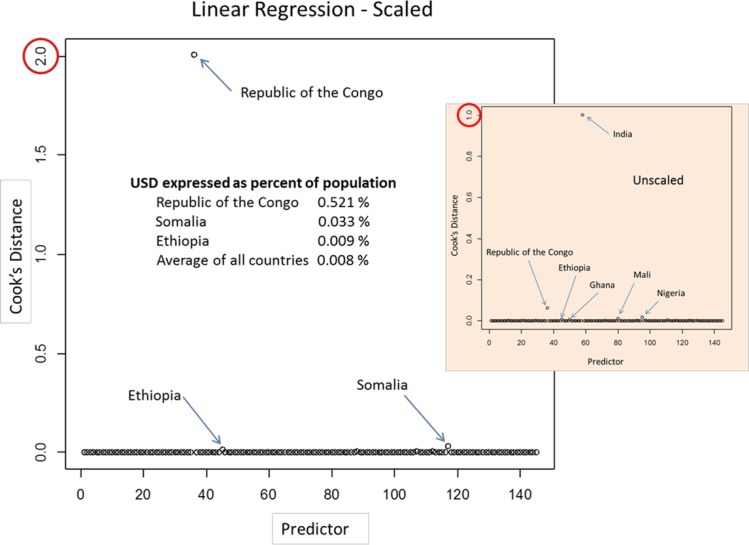
Cook’s Distance Outlier Plots.

We then scaled response by country population, which had sizable effects on both linear regression results and outlier detection. Fig 1 shows Cook’s Distances for both scaled USD response and unscaled (inset). Unscaled, India stands apart from other countries but upon scaling by population, People’s Democratic Republic of the Congo (DR Congo) emerges as a distant outlier, with Unsafe Sanitary Death Fraction (USDF) an order of magnitude greater than any other country.

Scaling yielded an increase in fraction of variance explained (adjusted R^2^ = 0.1165 for USD) over the unscaled response models and considerable improvements in statistical significance for PWTP, OIWS and UWS (e.g., B-H adjusted R^2^ = 0.0248, 0.0248 and 0.025 respectively) as shown in Table 2. The F-statistic is highly significant (0.0007) but relatively low in magnitude (4.165). Removing People’s Democratic Republic of the Congo from the data set had a pronounced effect on model fit as shown in Table 3. Adjusted R^2^ increased from 0.1165 to 0.6404 and the F-statistic increased to 43.45.

**Table 2:** Run002—All Countries (145) No transformations—Response is USDF.

	Estimate	Std. Error	t value	Pr(>|t|)	Bonferroni	H-B	B-H
(Intercept)	2.015811	0.778336	2.59	0.01063	0.07441	0.06832	0.024803
PWTP	-2.03844	0.781791	-2.607	0.01013	0.07091	0.06832	0.024803
OIWS	-2.04473	0.780244	-2.621	0.00976	0.06832	0.06832	0.024803
UWS	-1.92741	0.778235	-2.477	0.01447	0.10129	0.06832	0.025323
FBH	-0.00095	0.018033	-0.052	0.95826	1	1	0.95826
IS	0.017638	0.024723	0.713	0.47679	1	1	0.667506
HW	0.008148	0.054474	0.15	0.88131	1	1	0.95826

Residual standard error: 0.04104 on 138 degrees of freedom.

Multiple R-squared: 0.1533, Adjusted R-squared: 0.1165.

F-statistic: 4.165 on 6 and 138 DF, p-value: 0.0007.

**Table 3:** Run101– all countries except DRCongo (144) No transformations–Response is USDF.

	Estimate	Std. Error	t value	Pr(>|t|)	Bonferroni	H-B	B-H
(Intercept)	0.10243	0.09373	1.093	0.27643	1	1	0.44192
PWTP	-0.09487	0.09420	-1.007	0.31566	1	1	0.44192
OIWS	-0.09608	0.09403	-1.022	0.30868	1	1	0.44192
UWS	-0.07043	0.09361	-0.752	0.45311	1	1	0.52863
IS	-0.00817	0.00293	-2.793	0.00597	0.04179	0.04179	0.04179
FBH	-0.00527	0.00213	-2.48	0.01436	0.10050	0.08615	0.05025
HW	-0.00037	0.00642	-0.058	0.954	1	1	0.95400

Residual standard error: 0.004836 on 137 degrees of freedom.

Multiple R-squared: 0.6555, Adjusted R-squared: 0.6404.

F-statistic: 43.45 on 6 and 137 DF, p-value: < 2.2e-16.

The unadjusted p-values for IS and FBH became statistically significant at the .05 level (0.00597 and 0.01436 respectively). But note that the statistical significance levels reported for these results must be taken in proper context (residuals are not normally distributed); these models do not satisfy the conditions needed to legitimize the use of linear models [27, 34].

### Transformed Scaled Response

Transforming the data to achieve models that satisfy the conditions needed to apply ordinary least squares regression dramatically altered results in some important respects. Guided by Mosteller and Tukey’s bulging rule [28] we performed logit and power response transformations and removed the Democratic People’s Republic of the Congo from the data set. Response transformation, however, presents a complication in that some countries in the data set reported zero deaths for some responses. We examined two ways of handling these cases: (1) replace zero deaths with one death and modify scaled responses accordingly; and (2) include only countries with nonzero reported deaths (this reduces the sample size from the full complement of 145 countries to 122 for USDF; sample sizes for the other responses range from 121 to 139; see S1 File §1. Table 4 contains the power response transformed results for case 1. Table 5 shows the results for case 2. We see very large increases in adjusted R^2^ (to 0.7022, case 1 and to 0.8344 for case 2) and F-statistic (to 57.58, case 1 and to 102.6, case 3, both cases p < 2.2e-16). As summarized in S1 File §1, similar results occur for the other four responses.

**Table 4:** Run012– All Countries (145) Zero Deaths reset to 1—Power response transformation (USDF).

	Estimate	Std. Error	t value	Pr(>|t|)	Bonferroni	H-B	B-H
(Intercept)	2.68396	1.01101	2.655	0.00887	0.06209	0.05322	0.03105
PWTP	-1.91071	1.0155	-1.882	0.062	0.43403	0.31002	0.11286
OIWS	-1.88883	1.01349	-1.864	0.06449	0.45144	0.31002	0.11286
UWS	-1.77736	1.01088	-1.758	0.08093	0.56648	0.31002	0.11330
IS	-0.21028	0.03211	-6.548	1.06E-09	0.00000	0.00000	0.00000
FBH	0.01798	0.02342	0.768	0.44399	1.00000	0.44399	0.44399
HW	-0.10703	0.07076	-1.513	0.13266	0.92865	0.31002	0.15478

Residual standard error: 0.05331 on 138 degrees of freedom.

Multiple R-squared: 0.7146, Adjusted R-squared: 0.7022.

F-statistic: 57.58 on 6 and 138 DF, p-value: < 2.2e-16.

**Table 5:** Run057– Countries with nonzero response (122) Power response transformation (USDF).

	Estimate	Std. Error	t value	Pr(>|t|)	Bonferroni	H-B	B-H
(Intercept)	2.38023	0.90935	2.618	0.01	0.07034	0.06029	0.03517
PWTP	-1.69224	0.91201	-1.856	0.0661	0.46258	0.33042	0.11518
OIWS	-1.61025	0.91117	-1.767	0.0798	0.55888	0.33042	0.11518
UWS	-1.4921	0.9091	-1.641	0.1035	0.72428	0.33042	0.12071
IS	-0.17819	0.02696	-6.609	1.26E-09	0.00000	0.00000	0.00000
FBH	-0.03507	0.02	-1.753	0.0823	0.57589	0.33042	0.11518
HW	-0.13679	0.08893	-1.538	0.1268	0.88734	0.33042	0.12676

Residual standard error: 0.04267 on 115 degrees of freedom

Multiple R-squared: 0.8426, Adjusted R-squared: 0.8344

F-statistic: 102.6 on 6 and 115 DF, p-value: < 2.2e-16

The sole statistically significant predictor was IS (unadjusted p = 1.06e-09 for case 1 and p = 1.26e-09 for case 2). Later results will confirm the exceptional influence of IS. Note that neither of these models meet the criteria (most notably, normality of residuals) needed to justify the use of linear models.

### Predictor Transformation

**Fig 2:**
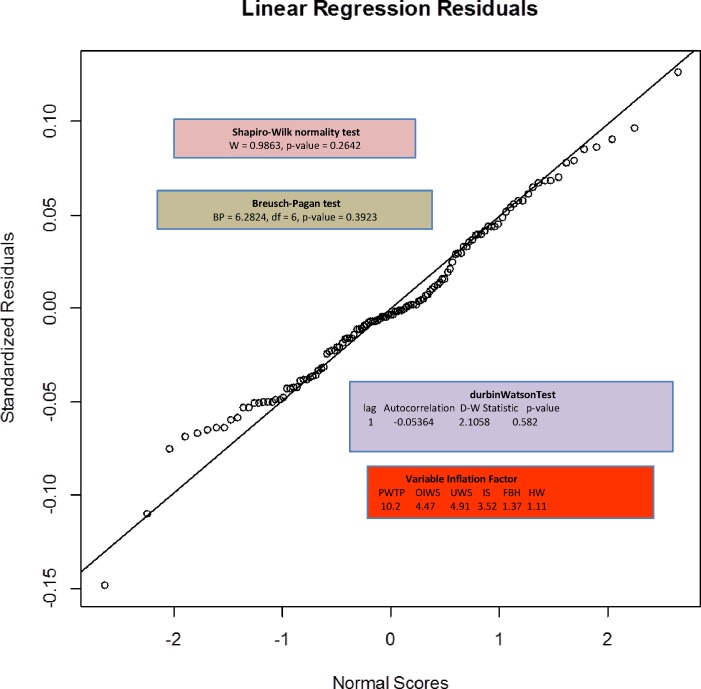
OLS Regression Results.

The next refinement in our model building involved transforming the six explanatory variables. Guided again by the bulging rule, we selected a square-root transformation and as before, transformed response (using a maximum likelihood Box Cox transformation [35]). Table 6 and Fig 2 show representative results for response = USDF, including only nonzero response countries with DR Congo removed.

**Table 6:** Run076– Countries with nonzero response and no DRCongo (121) Power response and square root predictor transformations (USDF).

	Estimate	Std.Error	t value	Pr(>|t|)	Bonferroni	H-B	B-H
(Intercept)	0.51629	0.07942	6.5	2.20E-09	1.54E-08	1.32E-08	7.69E-09
PWTP	-0.0297	0.05215	-0.57	0.57006	1.00000	0.57006	0.57006
OIWS	0.06585	0.0413	1.594	0.11363	0.79542	0.34089	0.15908
UWS	0.19281	0.04733	4.074	8.58E-05	0.00060	0.00043	0.00020
IS	-0.27231	0.04037	-6.744	6.67E-10	4.67E-09	4.67E-09	4.67E-09
FBH	-0.06739	0.02175	-3.098	0.00246	0.01719	0.00982	0.00430
HW	-0.11035	0.09532	-1.158	0.24939	1.00000	0.49879	0.29096

Residual standard error: 0.04656 on 114 degrees of freedom.

Multiple R-squared: 0.8606, Adjusted R-squared: 0.8533.

F-statistic: 117.3 on 6 and 114 DF, p-value: < 2.2e-16.

UWS, FBH and IS emerge as statistically significant, adjusted R^2^ is 0.8533, F-statistic is 117.3 and as shown in Fig 2, this model satisfies the diagnostic conditions required for using a linear model. Note that the null hypotheses for the Shapiro-Wilk, Breusch-Pagan and Durbin-Watson diagnostic tests are defined such that statistical significance is indicated by p-values that *exceed* a critical (e.g., 0.05) threshold.

The Variable Inflation Factor results (reflecting predictor collinearity) are, however, a reason to take note, especially for PWTP (but as discussed later, we have concerns about the validity and usefulness of this variable; others have expressed similar concerns [6]). Rules of thumb regarding levels of VIF becoming high enough such that linear regression results are compromised, range from 4 to about 10, but as described in [36] these rules are difficult to apply generally and need not invalidate the results.

**Fig 3:**
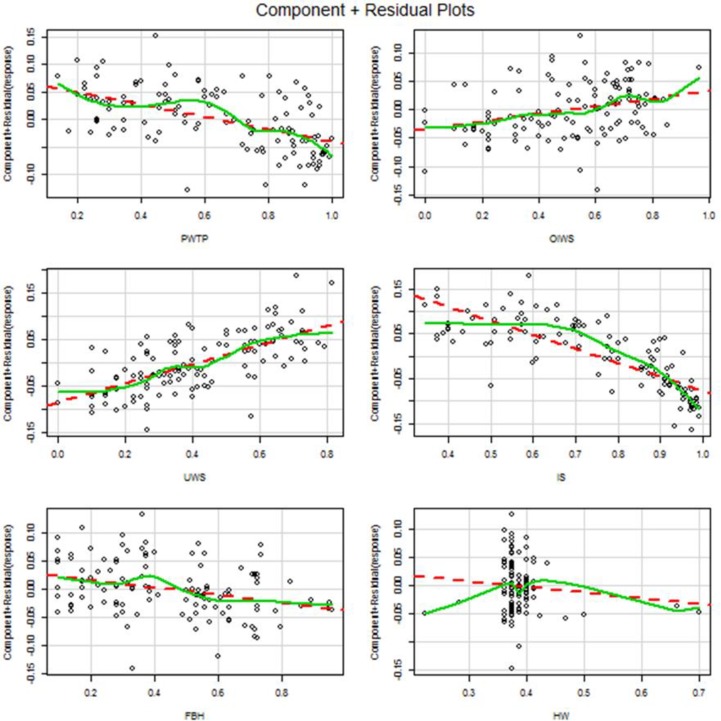
Component Plus Residual Plots.

Component-plus-residual plots for this model are shown in Fig 3 and reveal useful information regarding predictor contributions to model fit. HW in particular stands out as having very little explanatory power. IS and UWS clearly exhibit reasonably good fits, underscoring their dominant role as the most important explanatory variables.

We looked more carefully at relative variable importance by first computing Pratt importance scores shown in Table 7.

**Table 7:** Pratt Relative Variable Importance Scores.

Variable	Pratt Score	Lower 95% CI	Upper 95% CI
IS	0.4544	0.3214	0.5963
UWS	0.311	0.1638	0.4673
OIWS	0.087	-0.015	0.2087
FBH	0.0724	0.0274	0.128
PWTP	0.0609	-0.151	0.2598
HW	0.0144	-0.0103	0.0382

There is a pronounced break, dividing the predictors into most important (IS and UWS), somewhat important (FBH) and much less important predictors (OIWS, PWTP and HW), with the 95 percent confidence intervals of the scores for OIWS, PWTP and HW including zero. Another assessment of relative variable importance is possible through stepwise regression (using stepAIC and stepBIC). We found it notable that regardless of model performance criterion (AIC or BIC) and regardless of stepwise direction (forward, backward or two-way) the model shown in Table 8 was always identified as best. Moreover, this sub-setted model has about the same fraction of variance explained as its six-predictor parent, but has a considerably higher F statistic.

**Table 8:** Run096 –StepAIC Forward—Countries with nonzero response—no DRCcongo (121) Power response and square root predictor transformations (USDF).

	Estimate	Std. Error	t value	Pr(>|t|)	Bonferroni	H-B	B-H
(Intercept)	0.44334	0.04352	10.188	<2e-16	4.26E-17	4.26E-17	4.26E-17
IS	-0.28284	0.03929	-7.199	6.51E-11	3.25E-10	2.60E-10	1.63E-10
UWS	0.21294	0.03592	5.929	3.22E-08	1.61E-07	9.65E-08	5.36E-08
OIWS	0.08501	0.02528	3.363	1.04E-03	0.00522	0.00209	0.00131
FBH	-0.06508	0.02153	-3.023	3.08E-03	0.01542	0.00308	0.00308

Residual standard error: 0.0465 on 116 degrees of freedom.

Multiple R-squared: 0.8586, Adjusted R-squared: 0.8537.

F-statistic: 176 on 4 and 116 DF, p-value: < 2.2e-16.

Starting with the four predictor model, we then created a series of progressively smaller models that produced the results shown in Table 9 and serve to reinforce the remarkably influential roles of UWS and IS.

**Table 9:** Progressively Smaller Models.

	**Estimate**	**Std. Error**	**Pr(>|t|)**	**Bonferroni**	**H-B**	**B-H**
(Intercept)	0.4573	0.04356	< 2e-16	8.00E-16	8.00E-16	8.00E-16
IS	-0.31833	0.03628	2.98E-14	1.19E-13	8.94E-14	5.96E-14
UWS	0.25464	0.03602	1.67E-10	6.68E-10	3.34E-10	2.23E-10
OIWS	0.06328	0.02688	0.0204	0.0816	0.0204	0.0204
Adjusted R-squared: 0.8704		F-statistic: 247.2	p-value: <2.2E-16		Response power = 0.1636519	
	**Estimate**	**Std. Error**	**Pr(>|t|)**	**Bonferroni**	**H-B**	**B-H**
(Intercept)	0.20535	0.01582	< 2e-16	6.00E-16	6.00E-16	3.00E-16
UWS	0.45125	0.03337	< 2e-16	6.00E-16	6.00E-16	3.00E-16
OIWS	0.13999	0.03247	3.59E-05	0.0001077	3.59E-05	3.59E-05
Adjusted R-squared: 0.7806		F-statistic: 196.7	p-value: < 2e-16		Response power = 0.1192828	
	**Estimate**	**Std. Error**	**Pr(>|t|)**	**Bonferroni**	**H-B**	**B-H**
(Intercept)	0.69783	0.03703	< 2e-16	6.00E-16	6.00E-16	3.00E-16
IS	-0.49186	0.03207	< 2e-16	6.00E-16	6.00E-16	3.00E-16
OIWS	0.10222	0.0319	0.00178	0.00534	0.00178	0.00178
Adjusted R-squared: 0.8112		F-statistic: 237.4	p-value: < 2e-16		Response power = 0.1525925	
	**Estimate**	**Std. Error**	**Pr(>|t|)**	**Bonferroni**	**H-B**	**B-H**
(Intercept)	0.46334	0.039	< 2e-16	6.00E-16	6.00E-16	3.00E-16
IS	-0.34087	0.03467	< 2e-16	6.00E-16	6.00E-16	3.00E-16
UWS	0.26659	0.03545	1.71E-11	5.13E-11	1.71E-11	1.71E-11
Adjusted R-squared: 0.8665		F-statistic: 357.9	p-value: < 2e-16		Response power = 0.1834954	
	**Estimate**	**Std. Error**	**Pr(>|t|)**	**Bonferroni**	**H-B**	**B-H**
(Intercept)	0.44929	0.02059	< 2e-16	4.00E-16	4.00E-16	4.00E-16
OIWS	0.32892	0.03622	5.32E-15	1.06E-14	5.32E-15	5.32E-15
Adjusted R-squared: 0.4255		F-statistic: 82.47	p-value: 5.32E-15		Response power = 0.07328797	
	**Estimate**	**Std. Error**	**Pr(>|t|)**	**Bonferroni**	**H-B**	**B-H**
(Intercept)	0.15083	0.01409	<2e-16	4.00E-16	4.00E-16	2.00E-16
UWS	0.54896	0.03033	<2e-16	4.00E-16	4.00E-16	2.00E-16
Adjusted R-squared: 0.7481		F-statistic: 327.6	p-value: < 2e-16		Response power = 0.1538675	
	**Estimate**	**Std. Error**	**Pr(>|t|)**	**Bonferroni**	**H-B**	**B-H**
(Intercept)	0.73901	0.02024	<2e-16	4.00E-16	4.00E-16	2.00E-16
IS	-0.5486	0.02628	<2e-16	4.00E-16	4.00E-16	2.00E-16
Adjusted R-squared: 0.7981		F-statistic: 435.7	p-value: < 2e-16		Response power = 0.1791827	

Another test of model performance was made through k-fold cross validation. The results provide yet further evidence of the explanatory power of IS and UWS. When all six predictors are used, the 10-fold cross validation mean square error is 0.00226; when only IS and UWS are used, the corresponding prediction error increases only slightly to 0.00238.

### Categorical Response Transformation

The component-plus-residual plots shown in Fig 3 are indicative of a non-homogenous response which prompted us to reformulate response as a categorical variable comprised of USDF quartiles. We find the quartile country memberships intriguing. They are listed in Table 10.

**Table 10:** USDF Quartile Country Membership.

Quartile 1	Quartile 2	Quartile 3	Quartile 4
Algeria	Azerbaijan	RBhutan	Afghanistan
Argentina	RBangladesh	Bolivia	Angola
Armenia	Botswana	Comoros	Benin
Belarus	CaboVerde	Djibouti	BurkinaFaso
Brazil	Cambodia	Ethiopia	Burundi
Chile	DPRKorea	Gabon	Cameroon
China	DominicanRepublic	Gambia	CenAfrRep
Colombia	ElSalvador	Ghana	Chad
CostaRica	Fiji	Haiti	Côted'Ivoire
Cuba	Guatemala	RIndia	EquatorialGuinea
Ecuador	Guyana	Kiribati	Eritrea
Egypt	RIndonesia	LaoPDR	Guinea
Iran	Iraq	Lesotho	Guinea-Bissau
Jamaica	Mongolia	Liberia	Kenya
Jordan	Morocco	Malawi	Madagascar
Kazakhstan	RMyanmar	Micronesia	Mali
Kyrgyzstan	Nicaragua	Namibia	Mauritania
Malaysia	Panama	RNepal	Mozambique
Mexico	Paraguay	Pakistan	Niger
RussianFederation	Peru	Rwanda	Nigeria
Serbia	Philippines	SaoTomeandPrincipe	SierraLeone
Suriname	Samoa	Senegal	Somalia
Syria	SouthAfrica	SolomonIslands	SouthSudan
RThailand	RSriLanka	Swaziland	Sudan
Tunisia	Tajikistan	RTimor-Leste	Togo
Ukraine	Turkmenistan	Yemen	Uganda
Uzbekistan	Vanuatu	Zambia	URTanzania
Venezuela	VietNam	Zimbabwe	

Quartile 1, with the lowest USDF values, is comprised to a large extent of countries that are ex-socialist, Muslim, relatively oil rich, and/or recipients of large amounts of US aid. There are no sub-Saharan countries in this quartile. Quartile 4 with the highest USDF values contains many countries experiencing high levels of conflict (following [37]) and with the exception of Afghanistan, are all sub-Saharan.

### Consistency of Results for Different Mortality Responses

To illustrate the steps taken to achieve good model performance, our results thus far have focused on USDF response. An interesting and unexpected result was revealed in regression results for the remaining four responses in the WHO data set (Unsafe Water Deaths, Unsafe Hygiene Deaths, Unsafe Water and Sanitation Deaths and Unsafe Water, Sanitation and Hygiene Deaths). Whichever response was examined, the regression results remain remarkably consistent as shown in Table 11.

**Table 11:** Power response and square root predictor transformations—countries with nonzero response—no DRCongo.

**USDF**			**UHDF**			**UWDF**		
	Estimate	Pr(>|t|)		Estimate	Pr(>|t|)		Estimate	Pr(>|t|)
OIWS	0.0658	0.11363	OIWS	0.0232	0.540183	OIWS	0.001	0.9818
UWS	0.1928	8.58E-05	UWS	0.1512	0.000655	UWS	0.2337	1.03E-05
IS	-0.2723	6.67E-10	IS	-0.1043	0.006905	IS	-0.1277	0.0052
FBH	-0.0673	0.00246	FBH	-0.0699	0.000687	FBH	-0.1252	5.22E-07
HW	-0.1103	0.24939	HW	-0.086	0.196898	HW	-0.1368	0.0889
Adjusted R-squared: 0.8533			Adjusted R-squared: 0.7605			Adjusted R-squared: 0.8029		
F = 117.3; p-value: < 2.20E-16			F = 72.4; p-value: < 2.20E-16			F = 91.9; p-value: < 2.20E-16		
	**Wand SDF**				**WSandHDF**			
		Estimate	Pr(>|t|)			Estimate	Pr(>|t|)	
	PWTP	-0.0867	0.148739		PWTP	-0.1032	0.100637	
	OIWS	0.0115	0.804878		OIWS	-0.0081	0.865419	
	UWS	0.2432	1.05E-05		UWS	0.2508	1.50E-05	
	IS	-0.1832	0.000143		IS	-0.1698	0.000774	
	FBH	-0.104	4.67E-05		FBH	-0.0812	0.002078	
	HW	-0.139	0.09731		HW	-0.1393	0.087664	
	Adjusted R-squared: 0.816				Adjusted R-squared: 0.7929			
	F = 100.8; p-value: < 2.20E-16				F = 88.4; p-value: < 2.20E-16			

Across mortality responses, there is little change in variance explained and no change in which predictors are statistically significant at the 5% level. Similarly, fitted predictor estimates vary little with no discrepancies in sign.

## Discussion

Without response and predictor transformation, with number of deaths as response and including People’s Democratic Republic of the Congo (DRCongo), an OLS regression model explains essentially no observed variance and contains no statistically significant explanatory variables. Its F statistic is approximately one (and is not significant at the 0.05 level) providing further evidence that in this model, there are no significant predictors. These results occur for all modeled responses. When response is Unsafe Sanitation Death Fraction, dividing death count by country population (scaled response) we see a modest improvement in fit (R^2^ = 0.1165, F = 4.165) with three predictors (IS, UWS, PWTP) emerging as significant. DRCongo is an extreme outlier and also possesses high leverage [27] such that its removal increases R^2^ to 0.6404, F increases to 43.45 with a different subset of predictors becoming significant (IS and HW). Transforming response necessitates a modification to the data (zero deaths are computationally problematic) by either: (1) setting zero deaths to a small number (e.g., 1); or, (2) removing zero death countries from the analysis. We prefer approach 2 because we find zero death counts implausible. When a power transformation is performed for response USDF, R^2^ increases to 0.8344 with a concomitant increase in F to 102.6 (the corresponding results for approach 1 are 0.7022 and 57.58 as shown in §A in S1 File–run012). IS becomes the sole significant predictor. Transforming predictors (square root) yields a model with slightly improved R^2^ and F (0.8533, 117.3) and yet again a different set of significant predictors (IS, UWS, FBH).

We saw expected directions of association in the estimated predictor coefficients–negative for IS, FBH, HW and PWTP signifying decreases in USDF with increases in these predictors, and positive for UWS, but we also saw an unexpected and counterintuitive direction of association for OIWS (positive, USDF increases with OIWS). Concerns related to the interpretation and usefulness of OIWS as indicator are discussed in [6].

Experiments involving relative variable importance reaffirmed a now common theme that emerged in this work–IS and UWS are powerful explanatory variables, the remaining four predictors possessing far less explanatory power with PWTP and HW least useful. Regarding HW and FBH, this is perhaps not entirely unexpected given the caveat in [1]: “*Data based on limited country survey data*, *and modelled data provided for countries without survey information*. *These data should therefore be interpreted with caution*, *and provide indicative values only*.*”*

Another caveat is critically important. The results that we have presented for mortality responses plausibly do not apply when responses involve morbidity. More specifically, those predictors that we found uninfluential for predicting mortality-related responses may indeed be important when morbidity is the response. Diarrheal disease is a good example, where convincing evidence exists that hand washing for example (a predictor of essentially no use in predicting mortality) was found to be effective in reducing morbidity [14, 38]. This example again brings into play the important observation that piped water to premises doesn’t necessary mean safe water, with attendant consequences for morbidity and yet another outcome–malnutrition, in turn connected in a complex manner with both morbidity and mortality [14].

These results raise the following questions: (1) what are some of these predictors actually measuring? Do problems lie in their basic definitions? (2) Is the data for some of the predictors valid?; and, (3) are the responses reported in [1] truly measuring different phenotypes? The regression results reported in Table 11 (these are typical–other subsets of analyses look very similar across responses) are indifferent with respect to response-predictor associations. This seems at odds with definitions of what these responses and predictors are supposed to be measuring. We can’t fully explain this result, other than to speculate that the responses themselves are broadly capturing *something*, but are unable to resolve more specific predictor associations. A similar argument can perhaps be made for predictor non-specificity. From a somewhat broader perspective, it seems clear that all data–predictors and responses alike—is likely to be very heterogeneous. What might that unknown heterogeneity portend for data representativeness? More specifically, we refer, for example, to the potential for conflict to corrupt or otherwise distort reported data [11]. Is the result that Unsafe Sanitation Death Fraction for the People’s Democratic Republic of the Congo exceeds all other countries by an order of magnitude accurate, and if so, what is the role of decades of conflict and large-scale population movements?

These may have destroyed or made inaccessible a great deal of WASH-related infrastructure along with the health care systems that might have compensated for this loss. Of course, all of this is speculation, but the numbers suggest that we need to take specific contexts into account when we address these issues.

A second area of interest concerns those predictors that did not have a significant impact on mortality outcomes. In the data, the category of “piped water to premises” is the highest level of water supply, implying that it should have a significant positive effect. That is doesn’t suggests that the underlying reality is ambiguous. Piped water supplies may be intermittent. This may cause people to store water and the storage becomes the source of contamination. Or compromised distribution systems may allow contaminants to infiltrate. If people assume that piped water is clean water, they may cease to follow traditional safeguards such as boiling. In this context, research showing the degree to which improved water sources including piped water may be contaminated is particularly relevant [3, 31].

## Conclusions

Piped Water to Premises (PWTP) and handwashing (HW) had little value in predicting any mortality response. Good fitted model performance required: (1) the use of population-scaled death fractions as opposed to death totals; (2) transformed response (logit or power); and (3) predictor transformation (square root). The best models passed diagnostic tests for normality of residuals, linearity between predictors and response, and constant error variance, and exhibited remarkable performance given the heterogeneity of the countries involved and the complexity of the relationships between response and predictors. In the case of population-normalized Unsafe Sanitation Death fraction as response, the model explained about 85% of the observed variance in, with a high F-statistic and highly statistically significant predictor p-values. Two predictors—Improved Sanitation and Unimproved Water Sources–were most responsible for good model performance. Piped Water to Premises (PWTP) and hand washing (HW) had little value in explaining Unsafe Sanitation Death Fraction variance–results that were consistent across responses.

The fact that improved sanitation, closely followed by unimproved water source are always the most important predictors (regardless of mortality response) seems to us highly suggestive. In the absence of adequate blinded randomized controlled trials to determine the relative importance of water supply and sanitation or to understand how they interact, the strong signal in this data may provide some guidance. It suggests that efforts to provide clean water *in concert with* adequate sanitation will have the greatest impact and that adequate sanitation should be accorded a high priority in WASH policy.

The poor performance of other WHO predictors–specifically PWTP and HW–raises important questions. Previous research has suggested that PWTP, although it seems to imply good water quality, is not a guarantee of that [3,31]. Our results reinforce that conclusion, but, as we have suggested, its interpretation is not straightforward. HW, as a strictly behavioral variable, is particularly difficult to measure. Understandably, most of the field research in this area has been concerned with the effects of WASH interventions on health as an output. It may be that some research effort should also be directed to evaluating what these predictors are really able to tell us about WASH-related facilities as inputs.

There are many reasons to focus on the provision of clean water. Fetching water from any distance is a time-consuming burden that most often falls on women and girls. This reduces the time available for education and other productive activities. In some parts of the world, it puts the women and girls at direct physical risk. Further, fetching water over distances is likely to involve storage in the home, increasing the risk of recontamination even if the source water is reasonably clean.

If the goal is improved human health, however, our results suggest that the provision of clean water *by itself* is unlikely to achieve the desired outcome. Rather, the results indicate that the provision of clean water needs to be accompanied by improved sanitation in order for significant health benefits to be realized. We reiterate that while our focus in this paper is mortality, morbidity is a staggering consequence of inadequate water, sanitation and hygiene. Moreover, lower impact on mortality may not mean a similarly low impact on morbidity

That adequate sanitation plays an important role in health outcomes is not a new proposition. In the literature, the relative importance of water quality and sanitation in improving health outcomes has remained an open question [2, 9, 16, 38–45]. Our results suggest that a higher priority may need to be given to improved sanitation than has been the case. In this analysis, we have been able to show how important it is with a much higher degree of confidence. Further, our analysis suggests that we need to examine the meaning of the predictors on which we have been relying.

## Supporting Information

S1 File Supporting_Material_Overview. (DOCX)

S1 Data R_Source_Code.zip. (ZIP)
