# Supplementary material for: On the Identification of Associations between Five World Health Organization Water, Sanitation and Hygiene Phenotypes and Six Predictors in Low and Middle-Income Countries
Source: PLoS One. 2017 Jan 26;12(1):e0170451. doi: 10.1371/journal.pone.0170451 (PMC5268457; doi:10.1371/journal.pone.0170451)
Supplement: S1 File — (DOCX) [file pone.0170451.s001.docx]

**On the Identification of Associations between Five World Health Organization Water, Sanitation and Hygiene Phenotypes and Six Predictors in Low and Middle-Income Countries**

**Supporting Material**

**A - Compilation of Results**

Table 1 contains descriptions of all of the analyses performed, including:

- phenotypes other than Unsafe Sanitary Death Fraction
- two approaches for handling zero death counts: (1) reset zero deaths to one death and retain all 145 countries; and (2) remove zero death count countries resulting in 122 countries
- different phenotype transformations (no transformation, logit response transformation and power response transformation)
- no predictor transformation and square-root transformation

**Table 1. Master Results Compilation**

|  | **R^2** |
| --- | --- |
| run001 - all predictors (6) - no transformations - all countries (145) - USD | 0.01842 |
| run002 - all predictors (6) - no transformations - all countries (145) - USDF | 0.1165 |
| run003 - all predictors (6) - no transformations - all countries (145) - UHD | 0.01908 |
| run004 - all predictors (6) - no transformations - all countries (145) - UHDF | 0.107 |
| run005- all predictors (6) - no transformations - all countries (145) - UWD | 0.01988 |
| run006- all predictors (6) - no transformations - all countries (145) - UWDF | 0.1119 |
| run007- all predictors (6) - no transformations - all countries (145) - WandSD | 0.01866 |
| run008- all predictors (6) - no transformations - all countries (145) - WandSDF | 0.113 |
| run009- all predictors (6) - no transformations - all countries (145) - WSandHD | 0.01741 |
| run010- all predictors (6) - no transformations - all countries (145) - WSandHDF | 0.1126 |
|  |  |
| run011 - all predictors (6) - power response transformation - all countries (145) - zero deaths reset to 1 - USD | 0.6345 |
| run012 - all predictors (6) - power response transformation - all countries (145) - zero deaths reset to 1 - USDF | 0.7022 |
| run013 - all predictors (6) - power response transformation - all countries (145) - zero deaths reset to 1 - UHD | 0.4464 |
| run014 - all predictors (6) - power response transformation - all countries (145) - zero deaths reset to 1 - UHDF | 0.6077 |
| run015 - all predictors (6) - power response transformation - all countries (145) - zero deaths reset to 1 - UWD | 0.5238 |
| run016 - all predictors (6) - power response transformation - all countries (145) - zero deaths reset to 1 - UWDF | 0.6618 |
| run017 - all predictors (6) - power response transformation - all countries (145) - zero deaths reset to 1 - WandSD | 0.535 |
| run018 - all predictors (6) - power response transformation - all countries (145) - zero deaths reset to 1 - WandSDF | 0.6964 |
| run019 - all predictors (6) - power response transformation - all countries (145) - zero deaths reset to 1 - WSandHD | 0.5038 |
| run020 - all predictors (6) - power response transformation - all countries (145) - zero deaths reset to 1 - WSandHDF | 0.69 |
|  |  |
| run021 - all predictors (6) -logit response transformation - all countries (145) - zero deaths reset to 1 - USDF | 0.69 |
| run022 - all predictors (6) -logit response transformation - all countries (145) - zero deaths reset to 1 - UHDF | 0.5867 |
| run023 - all predictors (6) -logit response transformation - all countries (145) - zero deaths reset to 1 - UWDF | 0.6101 |
| run024 - all predictors (6) -logit response transformation - all countries (145) - zero deaths reset to 1 - WandSDF | 0.6738 |
| run025 - all predictors (6) -logit response transformation - all countries (145) - zero deaths reset to 1 - WSandHDF | 0.6677 |
|  |  |
| run026 - all predictors (6) -power response and square root predictor transformation - all countries (145) - zero deaths reset to 1 - USD | 0.6442 |
| run027 - all predictors (6) -power response and square root predictor transformation - all countries (145) - zero deaths reset to 1 - USDF | 0.6708 |
| run028 - all predictors (6) -power response and square root predictor transformation - all countries (145) - zero deaths reset to 1 - UHD | 0.4549 |
| run029 - all predictors (6) -power response and square root predictor transformation - all countries (145) - zero deaths reset to 1 - UHDF | 0.5909 |
| run030 - all predictors (6) -power response and square root predictor transformation - all countries (145) - zero deaths reset to 1 - UWD | 0.5312 |
| run031 - all predictors (6) -power response and square root predictor transformation - all countries (145) - zero deaths reset to 1 - UWDF | 0.6523 |
| run032 - all predictors (6) -power response and square root predictor transformation - all countries (145) - zero deaths reset to 1 - WandSD | 0.5437 |
| run033 - all predictors (6) -power response and square root predictor transformation - all countries (145) - zero deaths reset to 1 - WandSDF | 0.6836 |
| run034 - all predictors (6) -power response and square root predictor transformation - all countries (145) - zero deaths reset to 1 - WSandHD | 0.5136 |
| run035 - all predictors (6) -power response and square root predictor transformation - all countries (145) - zero deaths reset to 1 - WSandHDF | 0.6796 |
|  |  |
| run036 - all predictors (6) -power response and square root predictor transformation - all countries except DRCongo (144) - zero deaths reset to 1 - USD | 0.6442 |
| run037 - all predictors (6) -power response and square root predictor transformation - all countries except DRCongo (144) - zero deaths reset to 1 - USDF | 0.6891 |
| run038 - all predictors (6) -power response and square root predictor transformation - all countries except DRCongo (144) - zero deaths reset to 1 - UHD | 0.4439 |
| run039 - all predictors (6) -power response and square root predictor transformation - all countries except DRCongo (144) - zero deaths reset to 1 - UHDF | 0.6194 |
| run040 - all predictors (6) -power response and square root predictor transformation - all countries except DRCongo (144) - zero deaths reset to 1 - UWD | 0.5212 |
| run041 - all predictors (6) -power response and square root predictor transformation - all countries except DRCongo (144) - zero deaths reset to 1 - UWDF | 0.682 |
| run042 - all predictors (6) -power response and square root predictor transformation - all countries except DRCongo (144) - zero deaths reset to 1 - WandSD | 0.5343 |
| run043 - all predictors (6) -power response and square root predictor transformation - all countries except DRCongo (144) - zero deaths reset to 1 - WandSDF | 0.7201 |
| run044 - all predictors (6) -power response and square root predictor transformation - all countries except DRCongo (144) - zero deaths reset to 1 - WSandHD | 0.5034 |
| run045 - all predictors (6) -power response and square root predictor transformation - all countries except DRCongo (144) - zero deaths reset to 1 - WSandHDF | 0.72 |
|  |  |
| run046 - all predictors (6) - no transformations -countries with nonzero response (122) - USD | 0.005286 |
| run047 - all predictors (6) - no transformations -countries with nonzero response (122) - USDF | 0.1126 |
| run048 - all predictors (6) - no transformations -countries with nonzero response (137) - UHD | 0.01498 |
| run049 - all predictors (6) - no transformations -countries with nonzero response (137) - UHDF | 0.1075 |
| run050- all predictors (6) - no transformations -countries with nonzero response (136) - UWD | 0.01522 |
| run051- all predictors (6) - no transformations -countries with nonzero response (136) - UWDF | 0.1122 |
| run052- all predictors (6) - no transformations -countries with nonzero response (137) - WandSD | 0.01458 |
| run053- all predictors (6) - no transformations -countries with nonzero response (137) - WandSDF | 0.1126 |
| run054- all predictors (6) - no transformations -countries with nonzero response (139) - WSandHD | 0.01428 |
| run055- all predictors (6) - no transformations -countries with nonzero response (139) - WSandHDF | 0.1131 |
|  |  |
| run056 - all predictors (6) - power response transformation - countries with nonzero response (122) - USD | 0.5692 |
| run057 - all predictors (6) - power response transformation - countries with nonzero response (122) - USDF | 0.8344 |
| run058 - all predictors (6) - power response transformation - countries with nonzero response (137) - UHD | 0.4038 |
| run059 - all predictors (6) - power response transformation - countries with nonzero response (137) - UHDF | 0.7011 |
| run060- all predictors (6) - power response transformation - countries with nonzero response (136) - UWD | 0.4854 |
| run061- all predictors (6) - power response transformation - countries with nonzero response (136) - UWDF | 0.07608 |
| run062- all predictors (6) - power response transformation - countries with nonzero response (137) - WandSD | 0.5052 |
| run063- all predictors (6) - power response transformation - countries with nonzero response (137) - WandSDF | 0.7742 |
| run064- all predictors (6) - power response transformation - countries with nonzero response (139) - WSandHD | 0.4828 |
| run065- all predictors (6) - power response transformation - countries with nonzero response (139) - WSandHDF | 0.7432 |
|  |  |
| run066 - all predictors (6) - power response and square root predictor transformations - countries with nonzero response (122) - USD | 0.5727 |
| run067 - all predictors (6) - power response and square root predictor transformations - countries with nonzero response (122) - USDF | 0.8132 |
| run068 - all predictors (6) - power response and square root predictor transformations - countries with nonzero response (137) - UHD | 0.4056 |
| run069 - all predictors (6) - power response and square root predictor transformations - countries with nonzero response (137) - UHDF | 0.6904 |
| run070- all predictors (6) - power response and square root predictor transformations - countries with nonzero response (136) - UWD | 0.4917 |
| run071- all predictors (6) - power response and square root predictor transformations - countries with nonzero response (136) - UWDF | 0.7549 |
| run072- all predictors (6) - power response and square root predictor transformations - countries with nonzero response (137) - WandSD | 0.5089 |
| run073- all predictors (6) - power response and square root predictor transformations - countries with nonzero response (137) - WandSDF | 0.7678 |
| run074- all predictors (6) - power response and square root predictor transformations - countries with nonzero response (139) - WSandHD | 0.4839 |
| run075- all predictors (6) - power response and square root predictor transformations - countries with nonzero response (139) - WSandHDF | 0.7415 |
|  |  |
| run076 - all predictors (6) - power response and square root predictor transformations - countries with nonzero response - no DRCongo (121) - USDF | 0.8533 |
| run077 - all predictors (6) - power response and square root predictor transformations - countries with nonzero response - no DRCongo (137) - UHDF | 0.7605 |
| run078- all predictors (6) - power response and square root predictor transformations - countries with nonzero response - no DRCongo (136) - UWDF | 0.8029 |
| run079- all predictors (6) - power response and square root predictor transformations - countries with nonzero response - no DRCongo(137) - WandSDF | 0.816 |
| run080- all predictors (6) - power response and square root predictor transformations - countries with nonzero response - no DRCongo (139) - WSandHDF | 0.7929 |
|  |  |
| run081 - all predictors (6) - logit response and square root predictor transformations - countries with nonzero response - no DRCongo (121) - USDF | 0.8173 |
| run082 - all predictors (6) - logit response and square root predictor transformations - countries with nonzero response - no DRCongo (137) - UHDF | 0.6797 |
| run083- all predictors (6) - logit response and square root predictor transformations - countries with nonzero response - no DRCongo (136) - UWDF | 0.747 |
| run084- all predictors (6) - logit response and square root predictor transformations - countries with nonzero response - no DRCongo(137) - WandSDF | 0.7583 |
| run085- all predictors (6) - logit response and square root predictor transformations - countries with nonzero response - no DRCongo (139) - WSandHDF | 0.7289 |
| run096 - all predictors (6) - power response and square root predictor transformations - countries with nonzero response - no DRCongo (121) - USDF - variable subsetting |  |

**B – Basic Descriptive Statistics**

Descriptive statistics of the data follow and include predictor and response correlations, pairs plots and residual Normal Q-Q plots for the six predictors.

**Table 1. Correlation Matrices**


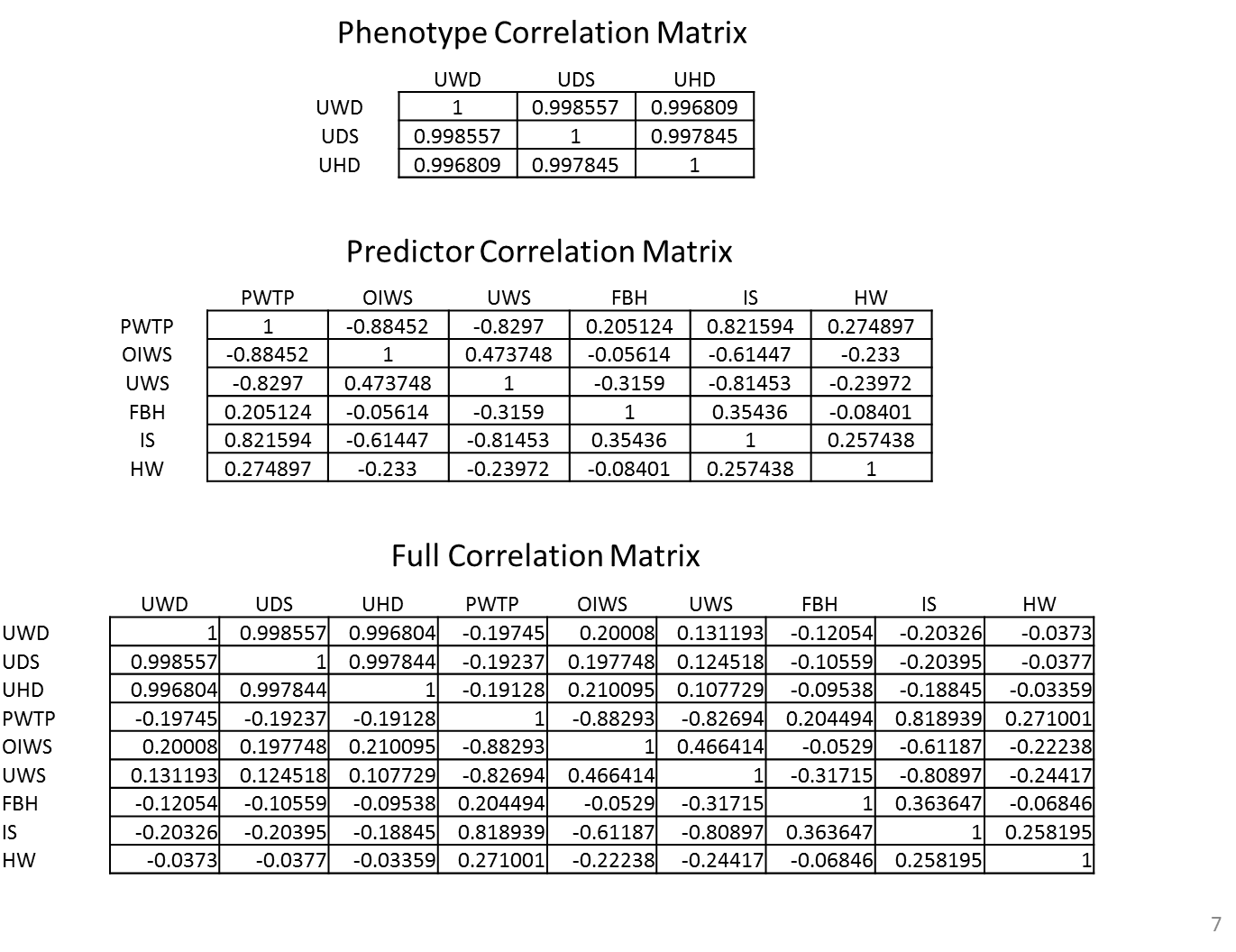


**Fig 1. Correlation Plot**


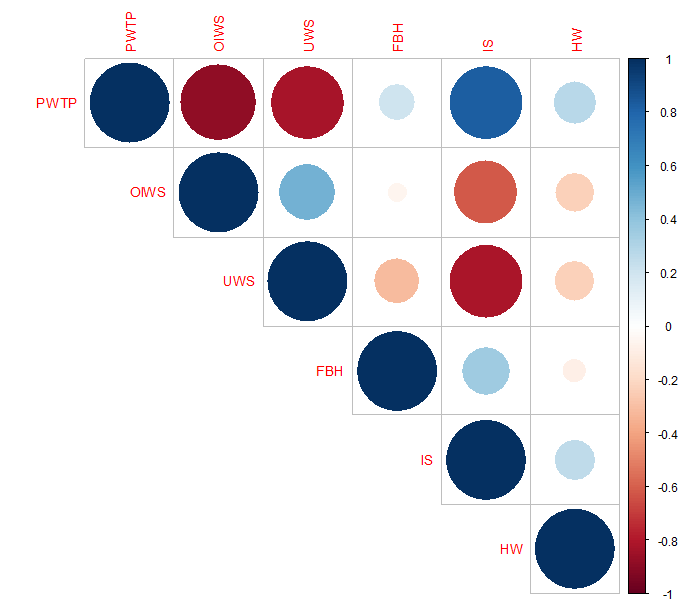


**Fig 2. Response and Predictor Scatterplot**


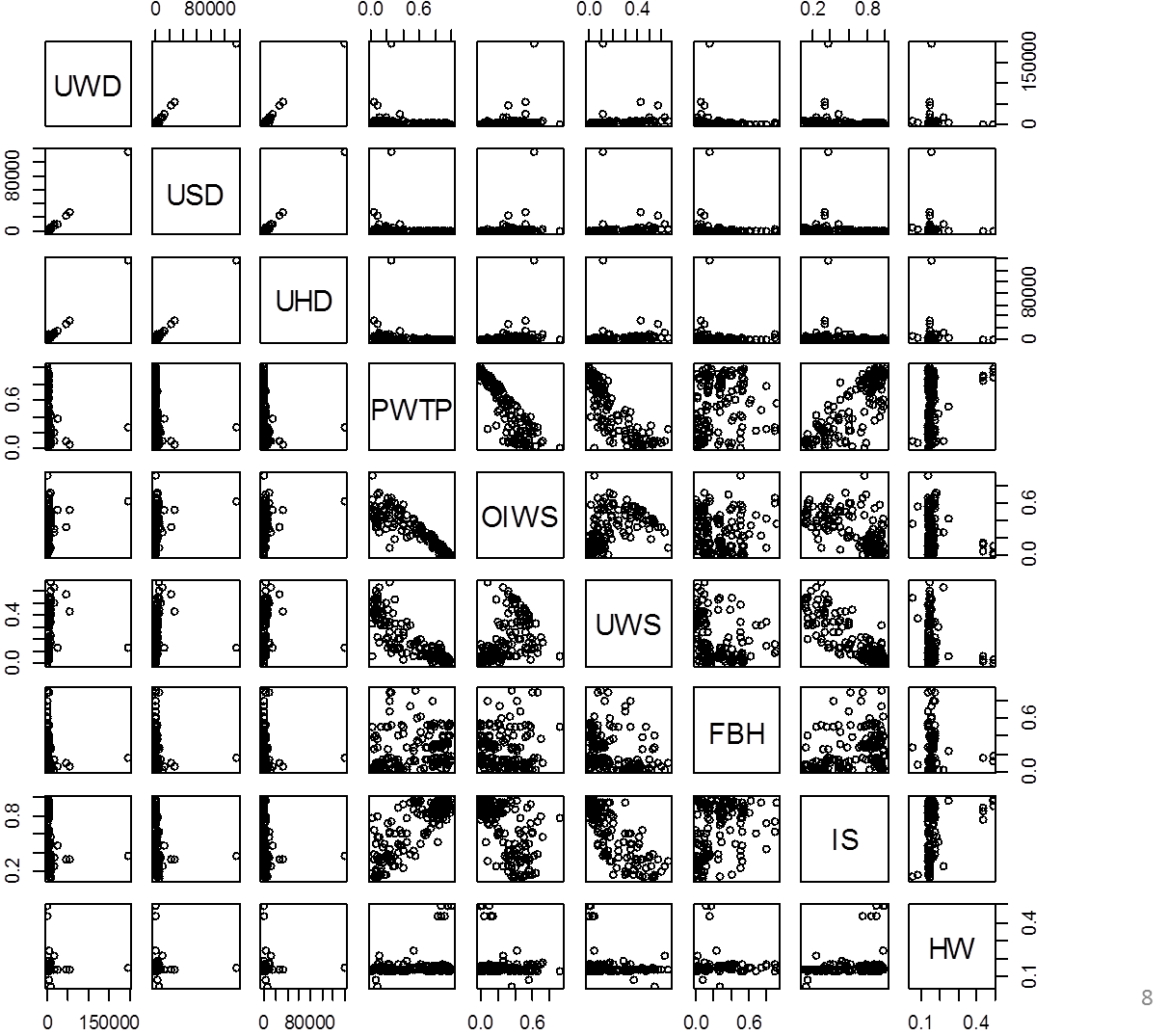


**Fig 3. Normal QQ Plots**


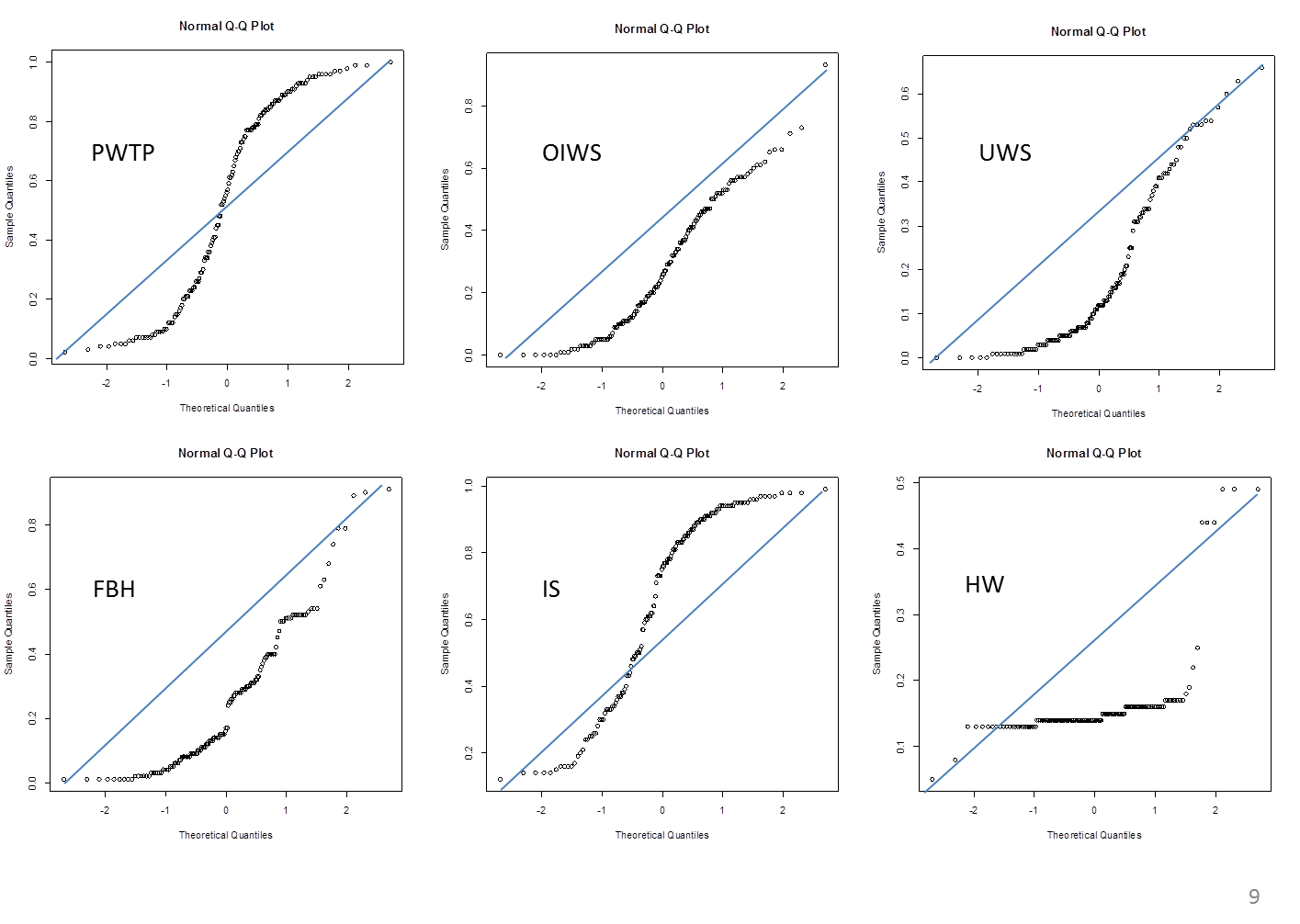


**C – R Source Code**

The file R_Source_Code.zip contains the relevant R code master files (runXXX.r); three templates (WHO_reanalysis.r, WHO_reanalysis_logit_transform.r, WHO_reanalysis_power_transform.r, which contain analysis-specific R code sourced by the master files); and, the raw input data files (either who_data_master_core_current.csv or who_data_master_core_current_zero_death_reset.csv). Executing a master file creates a subdirectory (runXXX) under containing: (1) a compilation of text output; (2) the saved R workspace image (runXXX.RData); (3) diagnostic plots in png format (avplots, component plus residual plots, effect plots, influence index plots, influence plot, marginal model plots, Normal Residual Plot, relaimpo plot, residual plots and spread level plot); and (4) partial dependence plots (one for each predictor (Partial Dependence Plot for FBH, partial Dependence Plot for HW, Partial Dependence Plot for IS, Partial Dependence Plot for OIWS, Partial Dependence Plot for PWTP and Partial Dependence Plot for UWS).
